# Base Materials’ Influence on Fracture Resistance of Molars with MOD Cavities

**DOI:** 10.3390/ma14185242

**Published:** 2021-09-12

**Authors:** Gabriela Ciavoi, Ruxandra Mărgărit, Liana Todor, Dana Bodnar, Magdalena Natalia Dina, Daniela Ioana Tărlungeanu, Denisa Cojocaru, Cătălina Farcaşiu, Oana Cella Andrei

**Affiliations:** 1 Department of Dental Medicine, Faculty of Medicine and Pharmacy, University of Oradea, 10 1st December Square, 410068 Oradea, Romania; gciavoi@uoradea.ro; 2 Department of Restorative Odontotherapy, Faculty of Dentistry, Carol Davila University of Medicine and Pharmacy, 37 Dionisie Lupu Str., 020021 Bucharest, Romania; ruxandra.margarit@gmail.com (R.M.); dana21bodnar@gmail.com (D.B.); 3 Department of Dental Techniques, Faculty of Midwifery and Nursing, Carol Davila University of Medicine and Pharmacy, 37 Dionisie Lupu Str., 020021 Bucharest, Romania; office@shinegrup.ro; 4 Department of Removable Prosthodontics, Faculty of Dentistry, Carol Davila University of Medicine and Pharmacy, 37 Dionisie Lupu Str., 020021 Bucharest, Romania; cella.andrei@gmail.com; 5 Independent Researcher, 020021 Bucharest, Romania; denisa.cojocaru96@yahoo.com; 6 Faculty of Dentistry, Department of Pedodontics, Carol Davila University of Medicine and Pharmacy, 37 Dionisie Lupu Str., 020021 Bucharest, Romania; catalina.farcasiu@yahoo.com

**Keywords:** mesial-occlusal-distal (MOD) cavities, fracture resistance, base materials

## Abstract

The aim of this study was to compare fracture resistance of teeth presenting medium-sized mesial-occlusal-distal (MOD) cavities using different base materials. Thirty-six extracted molars were immersed for 48 h in saline solution (0.1% thymol at 4 °C) and divided into six groups. In group A, the molars were untouched, and in group B, cavities were prepared, but not filled. In group C, we used zinc polycarboxylate cement, in group D—conventional glass ionomer cement, in group E—resin modified glass ionomer cement, and in group F—flow composite. Fracture resistance was tested using a universal loading machine (Lloyd Instruments) with a maximum force of 5 kN and a crosshead speed of 1.0 mm/min; we used NEXYGEN Data Analysis Software and ANOVA Method (*p* < 0.05). The smallest load that determined the sample failure was 2780 N for Group A, 865 N for Group B, 1210 N for Group C, 1340 N for Group D, 1630 N for Group E and 1742 N for Group F. The highest loads were 3050 N (A), 1040 N (B), 1430 N (C), 1500 N (D), 1790 N (E), and 3320 N (F), the mean values being 2902 ± 114 N (A), 972 ± 65 N (B), 1339 ± 84 N (C), 1415 ± 67 N (D), 1712 ± 62 N (E), and 2334 ± 662 N (F). A *p* = 0.000195 shows a statistically significant difference between groups C, D, E and F. For medium sized mesial-occlusal-distal (MOD) cavities, the best base material regarding fracture resistance was flow composite, followed by glass ionomer modified with resin, conventional glass ionomer cement and zinc polycarboxylate cement. It can be concluded that light-cured base materials are a better option for the analyzed use case, one of the possible reasons being their compatibility with the final restoration material, also light-cured.

## 1. Introduction

Fractures of posterior teeth with mesial-occlusal-distal (MOD) cavities restored with different materials can occur in mastication more frequently than those of healthy ones, proportionally with the quantity of hard dental tissues loss [1,2,3]. As restoration materials, those that adhere most to the dentin are the most recommended [4], considering that using them increases the resistance of the restored tooth [5,6]. A material used as a base for replacing lost dentine in a medium-sized cavity ensures a uniformly distributed load and tension across the filled tooth [7], especially in MOD cavities [8,9]. Among the most used base materials are glass ionomer cements, zinc polycarboxylate cements, zinc phosphate cements and resins. Nowadays, composite resins are preferred for restoring MOD cavities [10], offering good esthetics for an acceptable price [11,12]. Some authors mostly recommend replacing dentin with a glass ionomer cement or a flow composite as a base material [13,14]. Glass ionomer cements adhere to dental structures because they develop an ion-enriched interfacial zone with dentine [15]; they present a minimum contraction setting and less marginal infiltration than most composite resins [16]. Their mechanical properties are moderate [17], but their cariostatic effect and adhesion to dentin recommend them as base materials. Zinc polycarboxylate cements present mechanical and adhesive properties similar to glass ionomer cements [18]. Better, such properties are gained by glass ionomer cements enriched with resins. Flow composites used as base materials present the advantage of good adherence to the composite restoration material. They can be applied in layers of up to 4 mm and they adapt perfectly to the form of the prepared cavity. Studies reported that using flow composites as base materials determined a decrease of tensions in the restored tooth in class II cavities [19,20]; the recommended final restoration material for such a base is a special composite resin for posterior teeth [21]. The aim of this study was to compare the fracture resistance of teeth presenting medium sized mesial-occlusal-distal (MOD) cavities filled with the same composite resin, but having different base materials, in order to find out which base material is best to use for the long-term resistance of tooth in mastication. Medium sized mesial-occlusal-distal cavities are those affecting both the enamel and the dentin, in consequence needing two layers of filling material, but far enough from the pulp so they do not require pulp capping. The interactions of the materials used in the experiment with the dental structures, elasticity modulus and compression strength values are presented in Table 1.

## 2. Materials and Methods

### 2.1. Preparation of Teeth

**Figure 1:**
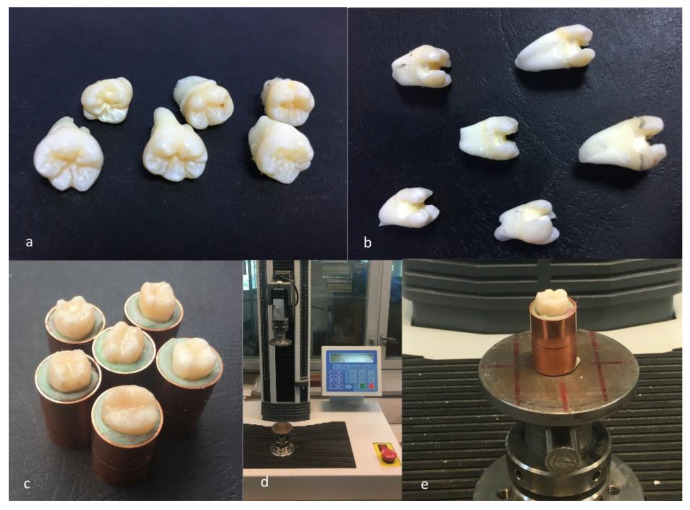
(**a**) Group of six molars unprepared; (**b**) group of six molars with MOD cavities; (**c**) group of samples prepared for testing; (**d**) universal loading machine; and (**e**) specimen before testing.

We used 36 molars, extracted for orthodontic purposes, with no previous cavities or fillings, that were collected from 4 private clinics and divided into six groups (Group A–F) of six teeth each (Figure 1a). They were cleaned by removing the remnant soft tissues and immersed for 48 h in saline solution containing 0.1% thymol at 4 °C, until the cavities were prepared, in order to avoid dehydration.

### 2.2. Preparation of Test Specimens

In the first of the six groups, the control group, the molars were kept untouched (Group A) (Figure 1b). In the teeth from the remaining five groups, mesial-occlusal-distal (MOD) medium sized cavities were prepared using the same burs at high speed, 30 identical round burs ISO 001/014 with a diameter of 1.4 mm and 30 identical cylindrical burs ISO 111/012 with a diameter of 1.2 mm, two new burs for each prepared molar; the cavities’ dimensions of 3.5 mm in width and 4.5 mm in height were verified using a digital caliper with an accuracy of 0.01 mm (Mitutoyo, Japan), cleaned and dried. In the second group, the medium-sized cavities were prepared, but were not filled at all, simulating a possible loss of the filling (Group B) (Figure 1c). In the other four groups, all final restorations were made with the same restoration material, using a universal composite (Charisma), but with four different types of base materials: Zinc polycarboxylate cement (zinc oxide with polyacrylic acid-metallic oxide—ZPC) for Group C, conventional glass ionomer cement (silicate glass powder and polyacrylic acid—GIC) for Group D, resin modified glass ionomer cement (hybrid materials of traditional glass ionomer cement with a small addition of light-curing resin—RMGIC) for Group E, and flow composite (flowable resin-based composites that are conventional composites with the filler loading reduced to 37–53% in volume—FC) for Group F (Table 2). The chemical composition of the materials used for the experiment is presented in Table 2. All fillings were done according to the manufacturer’s recommendations; the setting time was respected for all the materials used: 5–8 min for Adhesor carbofine, 6 min for Fuji IX and 20 s for the two light-cured materials.

For this experiment, the roots of the teeth were introduced in 36 identical cylindrical-shaped containers filled with a putty silicone material, in order to resiliently support them during the experiment and to mimic the oral cavity conditions (Figure 1c).

### 2.3. Fracture Resistance Test

Fracture resistance was tested using a universal loading machine (Lloyd Instruments, Segensworth, Fareham, UK) (Figure 1d); samples were subjected to vertical compression, with a maximum force of 5 kN and a crosshead speed of 1.0 mm/min until the fracture of the tooth; the results were recorded with NEXYGEN Plus 3 Data Analysis Software. A representative specimen is shown in Figure 1e. The graphics show data regarding the maximum fracture force values till the fracture of the most resistant specimen of each group. 

### 2.4. Statistical Analysis

Statistical analysis of obtained experimental values was performed using Microsoft Excel and ANOVA Method. For the variability of measured forces, mean values and standard deviations were analyzed. The level of significance was set at *p* < 0.05.

## 3. Results

**Figure 2:**
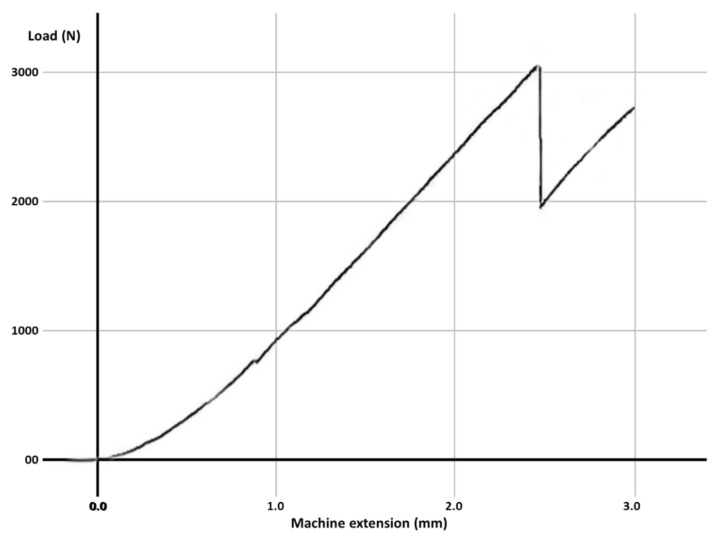
The maximum value of the force at which the most resistant molar from Group A failed.

**Figure 3:**
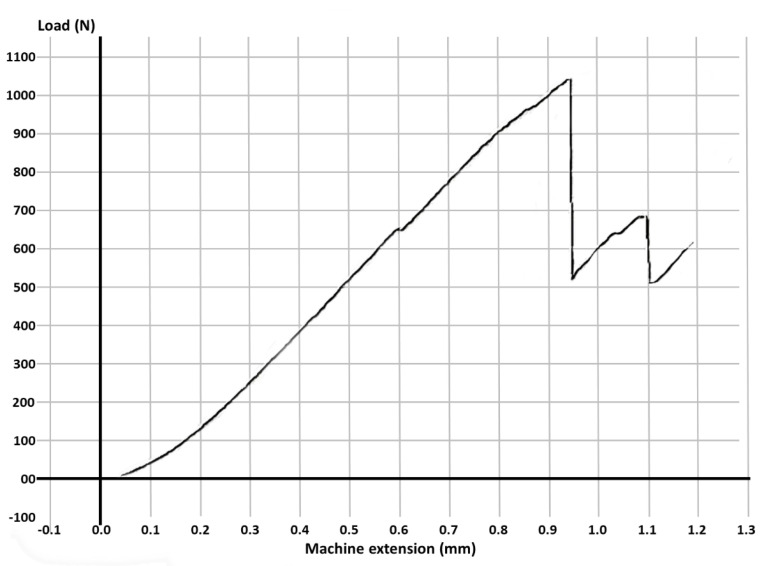
The maximum value of the force at which the most resistant molar from Group B failed.

**Figure 4:**
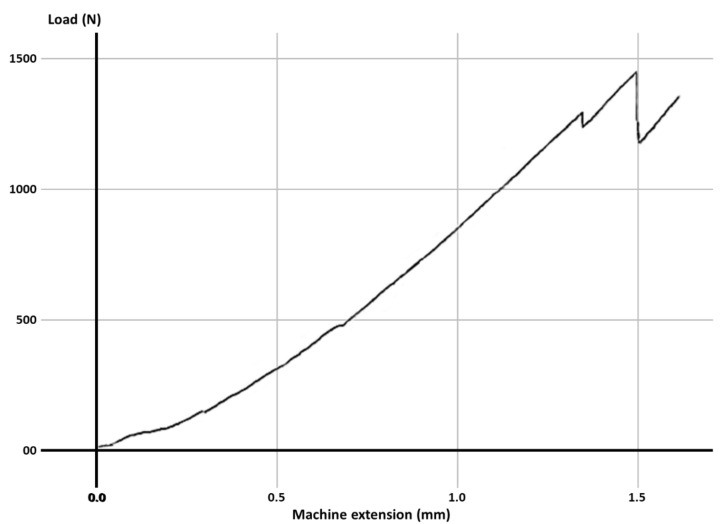
The maximum value of the force at which the most resistant molar from Group C failed.

**Figure 5:**
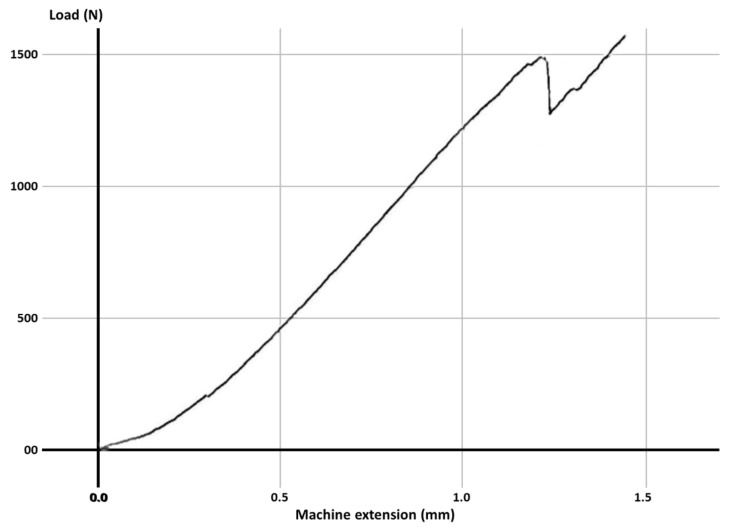
The maximum value of the force at which the most resistant molar from Group D failed.

**Figure 6:**
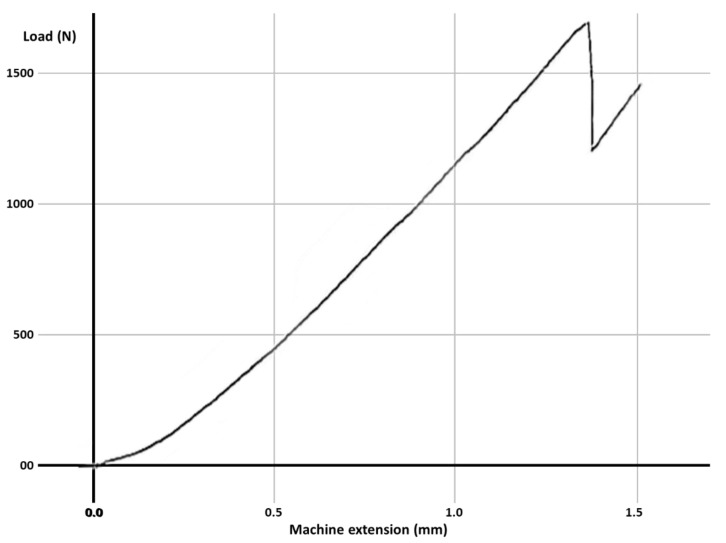
The maximum value of the force at which the most resistant molar from Group E failed.

**Figure 7:**
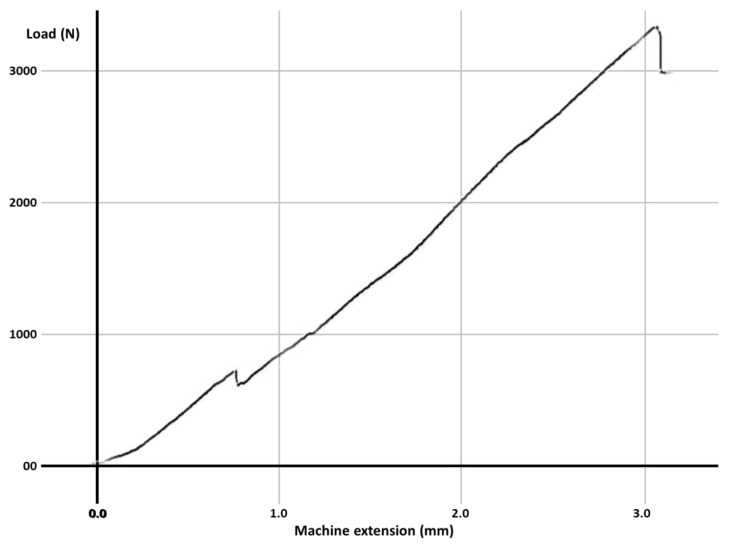
The maximum value of the force at which the most resistant molar from Group F failed.

**Figure 8:**
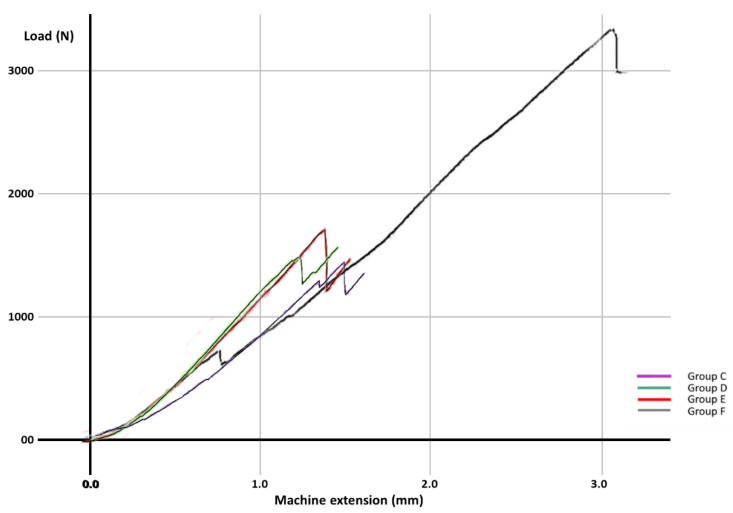
Result comparison for Groups C–F.

For each group, the test results for each molar, the mean fracture force, median and the standard deviation are expressed in Table 3. The graphs with the maximum value of the force in which the most resistant sample from each group failed is represented in Figure 2, Figure 3, Figure 4, Figure 5, Figure 6 and Figure 7. Group A, the control group, was stronger than all other groups, with a mean value of 2902 ± 114 N. Group B was weaker than all other groups, with a mean value of 972 ± 65 N. Group C and D were rather similar in terms of fracture resistance, with mean values of 1339 ± 84 N and 1415 ± 67 N. A more relevant difference was found between groups E and F, with mean values of 1712 ± 62 N and 2334 ± 662 N. In order to better compare the results for the four base materials that were used, the overlaid graphs of groups C–F are represented in Figure 8.

Statistical analysis using the ANOVA method in order to understand the relevance of the study revealed a *p* value of 0.000195, showing a statistically significant difference between Groups C–F restored with four different types of base materials (Table 4).

## 4. Discussion

Choosing the base material for medium-sized MOD cavities is difficult, because it can influence the long-term prognostic of the restored tooth. These cavities are involving both enamel and dentin; reducing the quantity of the dental tissues is a predisposing factor for fracture [1]. Studies reported that teeth with MOD cavities are losing their resistance in a proportion of 60%, compared to the non-prepared ones [22]. It has been reported that most recommended base materials for ensuring fracture resistance of the tooth are the ones presenting an elasticity modulus similar with the one of the dentin, such as composite resins [23,24], while the elasticity modulus of the zinc polycarboxylate cements and glass ionomer cements is smaller than that of the composite resins [25,26,27]. Some studies reported that using a base material with a low elasticity modulus presents the advantage of a higher deformation under occlusal forces, which reduces the fracture risk, while another study analyzing fracture resistance of non-vital teeth restored with different base materials showed that their different elasticity modulus did not influence fracture resistance of the teeth at all [28].

Other authors reported that conventional glass ionomer cement used as a base material had a positive influence on fracture resistance, teeth restored in such manner having a similar fracture resistance to the non-prepared ones [29,30]. Another study showed that glass ionomer cements used as base absorbed tensions generated during setting of the composite fillings [31]. Other authors showed that for non-vital teeth using glass ionomer cements as a base did not increase the fracture resistance [32,33], while another study concluded that using conventional glass ionomer cements as a base in MOD cavities can increase the resistance [34]. Eakle analyzed fracture resistance of adherent filling materials and showed that, although conventional glass ionomers have inferior mechanical properties compared to composite resins, using them as restoration materials did not decrease fracture resistance of the restored teeth [35]. Compared to conventional ones, new glass ionomers that are enriched with resins offer a better working time, due to the possibility to control the polymerization. The results of the study made by Oz et al. showed that the best fracture resistance was that of the teeth restored with MOD fillings that had bases of glass ionomer modified with resins, compared to conventional glass ionomers and flow composites [36]. Still, the results obtained by Taha et al. in a study on non-vital teeth having flow composite as a base showed that, using these materials, the fracture resistance of those teeth improved [37]; similarly, other studies observed the smallest fracture resistance for glass ionomer cements used as base, and the highest for flow composites [38,39,40,41]. In our study, the best fracture resistance was also obtained for the group having flow composite as a base, glass ionomer cements modified with resins being in the middle.

Using a base material under an adhesive composite filling increases the fracture resistance of the restored non-vital teeth [3,32,42]; still, the excessive thickness of the base has a negative influence on it [43]. Other studies showed that in case of teeth with massive loss of hard dental tissues the higher tensions appear in the remaining dental tissues and not to the interface between tooth and restoration, so the tooth can suffer a fracture [44,45]. In our in vitro experiment, the teeth were prepared in such manner that the resulting MOD cavities were medium-sized; within these limits, the highest fracture resistance was obtained using the flow composite as a base material. Additionally, our results showed that any restoration of teeth increased their fracture resistance, compared to the absence of the fillings. Further tests are necessary in order to assess how the results may change in case of larger, more profound cavities.

## 5. Conclusions

Regardless of the materials chosen for this study, the results showed that untouched molars (Group A) had the best fracture resistance, with much higher values obtained compared to the filled ones; also, the prepared but not filled at all molars (Group B) had the lowest values of all groups, showing that lost and not replaced fillings expose molars to significantly higher fracture risks. These results underline once more the importance of monitoring and prevention, especially in countries with poor or limited insurance systems. Within the limits of this study, for medium size mesial-occlusal-distal (MOD) cavities, filled with composite resins, the best base material that can be used in terms of fracture resistance proved to be the flow composite, followed by the glass ionomer modified with resin, and by the conventional glass ionomer cement. The smallest fracture resistance was obtained using zinc polycarboxylate cement as a base. It can be concluded that light-cured base materials are a better option for the analyzed use case, one of the possible reasons being their compatibility with the final restoration material, also light-cured.

## Figures and Tables

**Table 1:** Data regarding adhesion to the dental structures, elasticity modulus and compression strength.

Material	Adhesion	Modulus of Elasticity	Compression Strength
Adhesor carbofine (Spofa Dental)	Natural adhesion to the hard dental tissues	4.4 GPa	47 MPa
Fuji IX (GC)	Intrinsic adhesion to dentine and enamel, without the need for etching and bonding	8.3 GPa	220 MPa
Fuji II LC (GC)	Strong adhesion, excellent bond strength to teeth even in presence of saliva	5.33 GPa	245 MPa
Charisma flow (Heraeus Kulzer)	Adhesive for any bonding technique	14.3 GPa	325 MPa
Charisma (Heraeus Kulzer)	Adhesive for any bonding technique	8 GPa	325 MPa

**Table 2:** The materials used for teeth restoration.

Material	Purpose	Type	Chemical Composition
Adhesor carbofine (Spofa Dental)	Base	ZPC—zinc polycarboxylate cement	Zinc oxide, magnesium oxide, aluminum oxide, boric acid, acrylic acid, maleic anhydride, distilled water
Fuji IX (GC)	Base	GIC—glass ionomer cement	Alumino-silicate glass 95%, polyacrylic acid powder 5%
Fuji II LC (GC)	Base	RMGIC- Light-cured Resin Reinforced Glass Ionomer cement	Fluoro-alumino-silicate glass, polyacrylic acid 30–35%, distilled water 20–30%, 2HEMA 25–30%, initiator, urethan dymethylacrylate, camphorquinone
Charisma flow (Heraeus Kulzer)	Base	FC-Flowable resin-micro-hybrid flowable composite, Light-cured	multifunctional methacrylate monomers (EBADMA/TEGDMA); contains approximately 62% by weight or 38% by volume inorganic fillers such as Ba-AI-F silicate glass and SiO_2_. The filler particle size is between 0.005 μm and 5 μm.
Charisma (Heraeus Kulzer)	Final restoration	Universal hybrid composite with microparticles, Light-cured	BIS-GMA matrix; contains 64% filler by volume: barium aluminum fluoride glass (0.02–2 microns); colloidal silica −0.01–0.07 μm.

**Table 3:** The maximum force values at which the teeth in each of the six groups fractured.

Group	Mean (N)	Standard Deviation	Median	Fracture Force (N) for Each Specimen					
				1	2	3	4	5	6
A	2902	114	2889	2780	2795	2835	2943	3010	3050
B	972	65	988	865	930	972	1004	1025	1040
C	1339	84	1348	1210	1286	1315	1382	1413	1430
D	1415	67	1408	1340	1358	1372	1445	1478	1500
E	1712	62	1716	1630	1655	1698	1734	1765	1790
F	2334	662	2112	1742	1795	1855	2370	2925	3320

**Table 4:** ANOVA Method and *p* value.

ANOVA: Single Factor						
SUMMARY						
**Groups**	**Count**	**Sum**	**Average**	**Variance**		
C-ZPC	6	8036	1339.333	7126.267		
D-GIC	6	8493	1415.5	4563.1		
E-RMGIC	6	10,272	1712	3909.2		
F-FC	6	14,007	2334.5	438,639.5		
**ANOVA**						
**Source of Variation**	**SS**	**df**	**MS**	**F**	***p*-Value**	**F Crit**
Between Groups	3,682,527	3	1,227,509	10.80939		
Within Groups	2,271,190	20	113,559.5			
Total	5,953,717	23				

## Data Availability

Data is contained within the article.

## Sample Availability

Samples are not available from the authors.
